# Mechanisms of resistance to NAMPT inhibitors in cancer

**DOI:** 10.20517/cdr.2024.216

**Published:** 2025-04-16

**Authors:** Jansen Redler, Ariana E. Nelson, Christine M. Heske

**Affiliations:** Pediatric Oncology Branch, Center for Cancer Research, National Cancer Institute, Bethesda, MD 20892, USA.

**Keywords:** Nicotinamide phosphoribosyltransferase, inhibitor, resistance, cancer

## Abstract

A common barrier to the development of effective anticancer agents is the development of drug resistance. This obstacle remains a challenge to successful clinical translation, particularly for targeted agents. Nicotinamide phosphoribosyltransferase (NAMPT) inhibitors represent a clinically applicable drug class that exploits the increased dependence of cancer cells on nicotinamide adenine dinucleotide (NAD^+^), a coenzyme essential to metabolism and other cellular functions. NAMPT catalyzes the rate-limiting step in the NAD^+^ salvage pathway of mammalian cells and is overexpressed in numerous types of cancers. Preclinical research has demonstrated that pharmacological targeting of NAMPT may be an effective strategy against certain cancers, and while several early-phase clinical trials testing NAMPT inhibitors in refractory cancers have been completed, drug resistance is a concern. Preclinical work in a variety of cancer models has demonstrated the emergence of resistance to multiple NAMPT inhibitors through several recurrent mechanisms. This review represents the first article summarizing the current state of knowledge regarding the mechanisms of acquired drug resistance to NAMPT inhibitors with a particular focus on upregulation of the compensatory NAD^+^ production enzymes nicotinate phosphoribosyltransferase (NAPRT) and quinolinate phosphoribosyltransferase (QPRT), acquired mutations in NAMPT, metabolic reprogramming, and altered expression of the ATP-binding cassette (ABC) efflux transporter ABCB1. An understanding of how these mechanisms interact with the biology of each given cancer cell type to predispose to the acquisition of NAMPT inhibitor resistance will be necessary to develop strategies to optimize the use of these agents moving forward.

## INTRODUCTION

**Figure 1:**
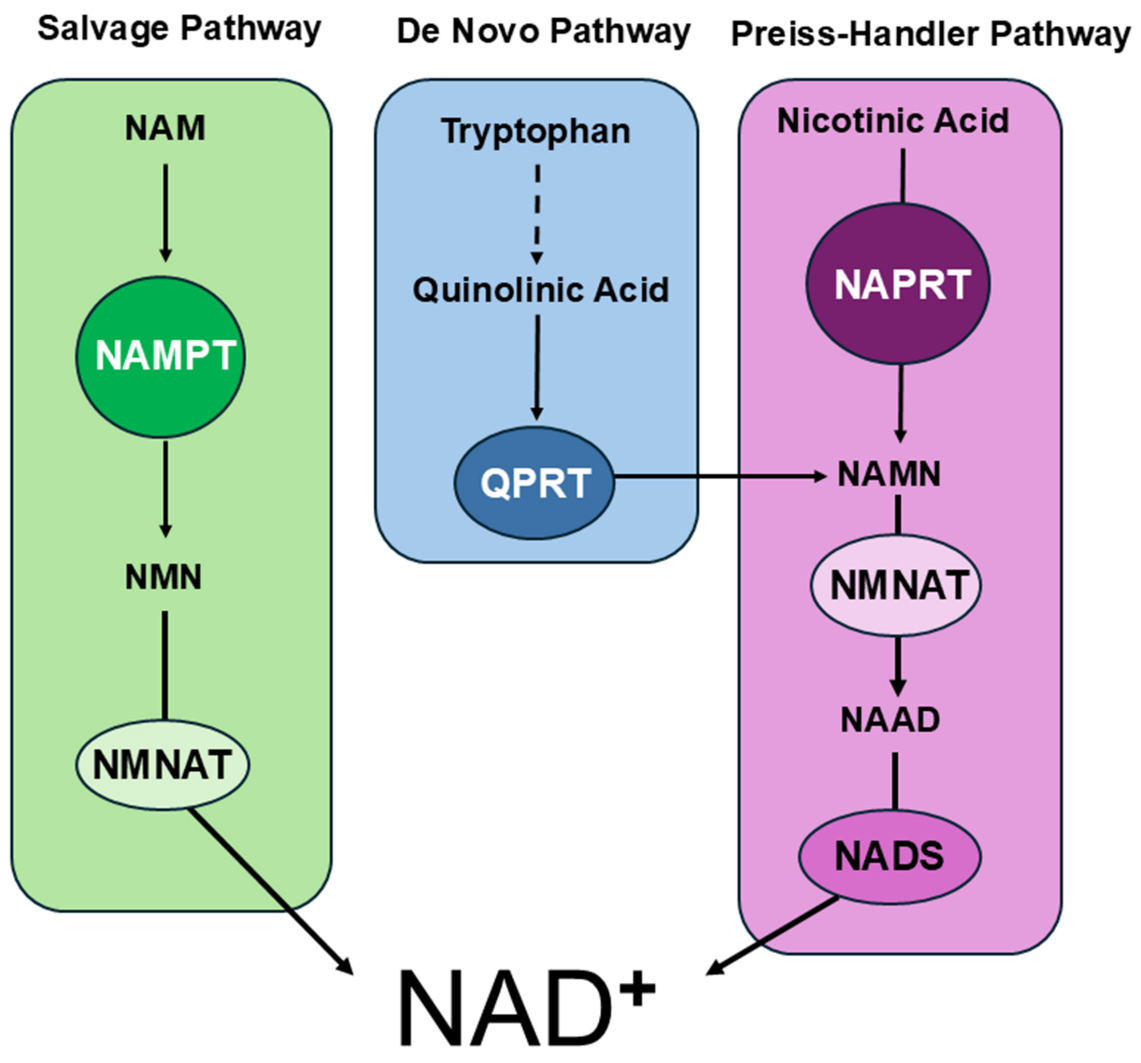
The three redundant NAD^+^ synthesis pathways: salvage (green), *de novo* (blue) and Preiss-Handler (purple). NAD^+^: Nicotinamide adenine dinucleotide.

Nicotinamide adenine dinucleotide (NAD^+^) is a critical cellular metabolite important for a wide range of cellular processes including energy production, DNA synthesis and repair, sirtuin function, and redox balance^[1]^. NAD^+^ generation primarily occurs through three key pathways: de novo synthesis initiated from cellular uptake of tryptophan, a salvage pathway that recycles nicotinamide (NAM) to NAD^+^, and conversion of nicotinic acid (NA, also known as niacin or vitamin B3) to NA mononucleotide (NAMN) by the enzyme nicotinate phosphoribosyltransferase (NAPRT1), which is then converted to NAD^+^ via the Preiss-Handler pathway NAD^+^^[2,3]^. In the salvage pathway, nicotinamide phosphoribosyltransferase (NAMPT) is the key enzyme, as it catalyzes the rate-limiting step in the biosynthesis of NAD^+^ [Figure 1]. NAMPT is primarily located in the cytoplasm and nucleus and is ubiquitously expressed in all tissues, although bone marrow, liver, and muscle, which have high energy requirements, have higher levels^[4,5]^. NAMPT is also secreted extracellularly (eNAMPT), where it has been shown to act as a pro-inflammatory cytokine, corresponding to tumor aggressiveness and poor prognosis in certain types of cancer^[6,7]^.

Many cancer cells are characterized by an increased demand for NAD^+^ relative to normal cells as a result of increased metabolic requirements for proliferation and growth^[8]^. Multiple hematologic and solid malignancies are characterized by NAMPT overexpression, including lymphoma, breast cancer, gastric cancer, and bladder cancer^[9,10]^. In some settings, overexpression of NAMPT correlates with increased tumor aggressiveness and poorer prognosis^[9,11]^. Increased access to NAD^+^ facilitated by NAMPT may directly increase cellular energy metabolism via increased activity of NAD^+^-dependent glycolytic enzymes, including glyceraldehyde-3-phosphate dehydrogenase (GAPDH) and lactate dehydrogenase (LDH)^[12]^. Indirectly, NAMPT may be involved in metabolic reprogramming via regulation through c-MYC^[13,14]^. NAMPT has also been linked to non-metabolic oncogenic signaling pathways; in some cases, oncogenic factors regulate the expression and activity of NAMPT^[15]^, while in others, *NAMPT* regulates the activity of oncogenic signaling pathways^[16]^.

Recognizing that there may be therapeutic benefits of limiting NAD^+^ in cancer cells, several pharmacological inhibitors of NAMPT have been developed. These include CHS-828 (also known as GMX1778)^[17]^, its more water-soluble prodrug GMX1777^[18,19]^, FK866 (also known as APO866 and WK175)^[20]^, GNE-617^[21]^, A-1293201^[22,23]^, KPT-9274^[24]^, and OT-82^[25]^. A number of these agents have entered clinical development and early phase study [Table 1]^[26-32]^. While phase 1 trials testing CHS-828, FK866, and GMX1777 were completed, and a phase 2 trial testing FK866 was completed, further development of most compounds was discontinued due to dose-limiting hematologic toxicity and limited efficacy as single agents, which primarily resulted in stable disease^[4]^. Several NAMPT inhibitors, namely KPT-9274 and OT-82, remain under clinical investigation.

**Table 1:** Clinical trials testing NAMPT inhibitors

**Drug**	**NCT number**	**Phase**	**Study population**	***n***	**Dosing schedule**	**Outcome/status**	**Ref.**
CHS-828	n/a	I	Advanced solid tumors	16	Oral dose days 1-5 q 28 days	RP2D defined	[26]
CHS-828	n/a	I	Advanced solid tumors	37	Oral dose once weekly q 21 days	RP2D defined	[27]
CHS-828	NCT00003979	I	Advanced solid tumors	7	Oral dose once weekly × 3 weeks q 28 days	RP2D not reached Terminated early due to halted drug development	[28]
FK866/APO866	n/a	I	Advanced solid tumors	24	Continuous 96-hour IV infusion q 21 days	RP2D defined	[29]
FK866/APO866	NCT00435084	I/II	B-cell CLL		Continuous 96-hour IV infusion q once	No results available	
FK866/APO866	NCT00431912	II	Cutaneous T-cell lymphoma	14	Continuous 96-hour IV infusion q 28 days	Of 14 patients, 1 PR, 6 SD Terminated early due to lack of efficacy	[30]
FK866/APO866	NCT00432107	II	Melanoma		Continuous 96-hour IV infusion q 28 days	No results available	
GMX1777	NCT00457574	I	Refractory solid tumors and lymphomas	19	Continuous 24-hour IV infusion q 21 days	RP2D defined	[31]
GMX1777 + TMZ	NCT00724841	I/II	Metastatic melanoma		IV infusion over 3 h on day 1, days 1 and 3, or days 1, 3 and 5 q 28 days	No results available	
KPT-9274/ATG-019 +/- Niacin ER	NCT04281420	I	Advanced solid tumors or NHL		Oral dose QoD3/week	No results available Terminated early due to change in drug development strategy	
KPT-9274/ATG-019 +/- Niacin ER +/- Nivolumab	NCT02702492	I	Advanced solid tumors or NHL	14	Oral dose QoD3/week	Partial results available - RP2D not reached at the time of reporting Terminated early	[32]
KPT-9274/ATG-019	NCT04914845	I/II	Relapsed/refractory AML		Oral dose QoD3/week	No results available Recruiting	
OT-82	NCT03921879	I	Relapsed/refractory lymphoma		Oral dose days 1-3 q week	No results available Recruiting	

NAMPT: Nicotinamide phosphoribosyltransferase; q: every; RP2D: recommended phase 2 dose; CLL: chronic lymphocytic leukemia; PR: partial response; SD: stable disease; TMZ: temozolomide; ER: extended release; NHL: non-Hodgkin’s lymphoma; QoD3/week: every other day three times per week; AML: acute myeloid leukemia.

An additional challenge to the development of NAMPT inhibitors, as with other targeted inhibitors, has been the potential for drug resistance. Resistance to therapeutics may be classified as either intrinsic resistance, where cells never respond to the therapy, or acquired resistance, which develops after initial responsiveness to therapy^[33]^. Acquired resistance may occur for a variety of reasons, including acquired changes to specific drug targets or regulatory pathways, activation of bypass pathways, rewiring of cellular metabolic pathways, alterations in cell death pathways, and/or enhanced DNA repair^[34]^.

**Figure 2:**
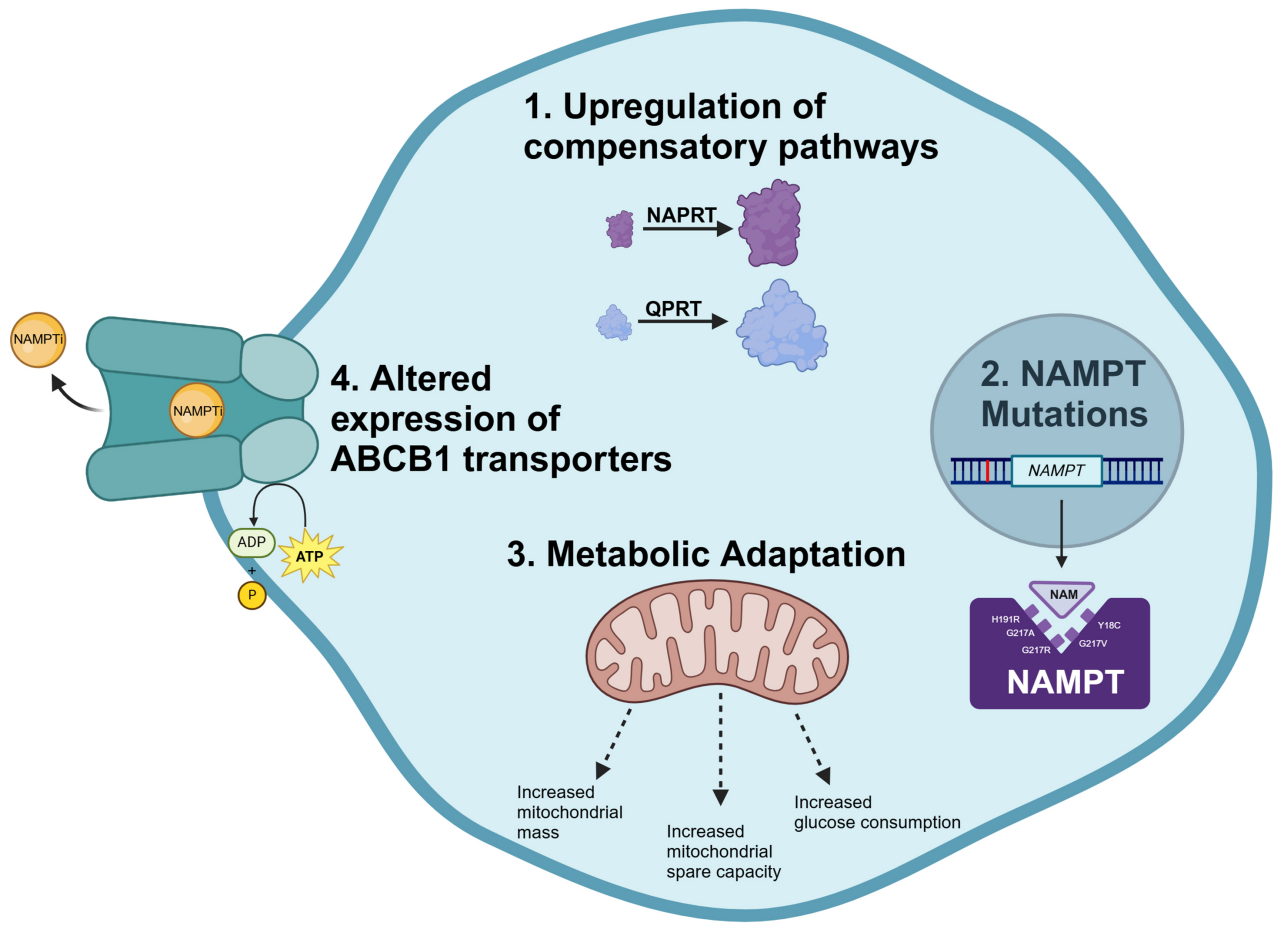
Mechanisms of NAMPT inhibitor resistance. Created with BioRender.com. NAMPT: Nicotinamide phosphoribosyltransferase.

In addition to the mechanisms occurring at a cellular level, factors such as insufficient intracellular drug accumulation within the tumor, inability of the drug to be metabolized to its optimal pharmacokinetic state, and changes in the absorption, distribution, metabolism, or excretion of a drug may also contribute to clinical drug resistance^[35]^. Given the high flexibility of cancer cells to adapt to new external pressures, including inhibition of critical targets, it is key to understand drug resistance by examining the underlying mechanisms so they may be potentially exploited therapeutically. Here, we summarize the current state of the science on NAMPT inhibitor resistance in cancer, focusing on altered regulation of compensatory NAD^+^ synthesis pathways, drug target mutations, metabolic adaptations, and drug transporters [Figure 2].

## ALTERATIONS IN REDUNDANT NAD^+^ SYNTHESIS PATHWAYS

As NAMPT is the rate-limiting enzyme in one of three redundant NAD^+^ biosynthetic pathways, alternations in the expression and/or activity of the other pathway-specific rate-limiting NAD^+^ synthesis enzymes have been investigated as potential mechanisms of resistance^[3,36,37]^. To determine how particular cancer cells may prioritize each NAD^+^ biosynthetic route, an analysis of more than 7,000 tumors and 2,600 matched normal samples, spanning 19 tissue types, was conducted and concluded that tumors arising from normal tissues with increased *NAPRT* expression typically exhibit amplification of *NAPRT*, suggesting increased reliance on the Preiss-Handler pathway for NAD^+^ production. Conversely, tumors originating from tissues lacking *NAPRT* expression were shown to be reliant on the salvage pathway in genetic knockout experiments^[38]^. Variable enzyme expression may play a role in tissue-specific sensitivity and resistance to NAMPT inhibition in cancer. One study examining the role of quinolinate phosphoribosyltransferase (QPRT) and NAPRT reported that when the substrates for these pathways are present in cells with these enzymes, NAMPT inhibition can be circumvented^[23]^.

Indeed, there is evidence that in some tumor types, elevated *NAPRT* expression may be associated with insensitivity to NAMPT inhibition. In xenograft models of ovarian carcinoma, silencing *NAPRT* or inhibiting the enzyme with 2-hydroxinicotinic acid resulted in sensitization to FK866; similarly, overexpression of *NAPRT* induced resistance in these models, supporting the idea that *NAPRT* amplification and overexpression represent a mechanism of resistance to NAMPT inhibitors^[39]^. Currently, there are few NAPRT inhibitors, none of which have reached the clinic. NAPRT inhibitory activity has been noted to be a feature of several compounds, such as 2-hydroxynicotinic acid (2-HNA), as well as several non-steroidal anti-inflammatory drugs (NSAIDs) such as flufenamic acid, mefenamic acid, and phenylbutazone^[40,41]^. In preclinical studies, exposure of NAPRT-positive ovarian and pancreatic cancer cells to 2-HNA sensitized them to NAMPT inhibitors and recapitulated the effect of *NAPRT* silencing. However, 2-HNA remains the only reported NAPRT inhibitor with demonstrated anticancer activity^[42]^. Due to its low potency and poor aqueous solubility, 2-HNA is not a viable clinical candidate^[42]^. More recently, two new enzymatic inhibitors of NAPRT were identified through a high-throughput in silico screening of a chemical library containing more than 500,000 compounds: compound 8 (4-hydroxynicotinic acid), which acts as a competitive NAPRT inhibitor, and compound 19, which acts as an uncompetitive NAPRT inhibitor. Among those two inhibitors, compound 8 was most effective at sensitizing the NAPRT-proficient ovarian cancer cell line OVCAR-5 to FK866^[42]^. A second approach using structure-based computation to identify NAPRT inhibitors isolated IM 29, a compound with a 1,3-benzodioxole structural backbone, as a lead compound^[43]^. Each of these newly described NAPRT inhibitors requires concentrations in the high micromolar-to-millimolar range to be effective^[44]^, underscoring the fact that efforts to move NAPRT targeting forward are still very early.

Similar to the findings on NAPRT, studies have shown that *QPRT* upregulation may also permit cells to bypass the salvage pathway and overcome NAMPT inhibition. Using a HT1080 cell line engineered to be resistant to GMX1778 (HT1080-GMX), the expression of 32 proteins involved in NAD^+^ biosynthesis and consumption was analyzed in comparison to the parental cell line. While no differences in *NAMPT* or *NAPRT* expression were observed in the resistant cell line compared to the parental cell line, *QPRT* was found to be overexpressed in HT1080-GMX cells. Furthermore, the cytotoxic effects of the NAMPT inhibitors GMX1778 and FK866 could be remedied by exposing HT1080 cells that reliably overexpress *QPRT* to exogenous quinolinic acid^[23]^. A second study using the T cell acute lymphoblastic leukemia cell line CCRF-CEM demonstrated that QPRT activity was modestly but significantly increased in NAMPT inhibitor-resistant cell lines compared to parental cells. To test whether CCRF-CEM NAMPT inhibitor-resistant cells could use tryptophan as an alternative NAD^+^ precursor, the activity of JPH203, an inhibitor of the L-type amino acid transporter 1 (LAT1) which blocks tryptophan uptake, plus FK866 was evaluated. Co-treatment of the resistant cell line with JPH203 and FK866 resulted in significant decreases in both NAD^+^ and ATP in the NAMPT inhibitor-resistant cell line^[45]^. In contrast, another study exploring FK866-resistant colorectal cells demonstrated that *QPRT* levels in parental and resistant cell lines were similar^[46]^. Currently, there are no reported inhibitors of QPRT, thus limiting the therapeutic applications of these findings. Taken together, these data demonstrate that both intrinsic and acquired alterations in the de novo and Preiss-Handler pathways for NAD^+^ production may act as bypass mechanisms contributing to NAMPT inhibitor resistance and that further development of strategies to target these mechanisms may be necessary to optimize the translation of NAMPT inhibitors.

## MUTATIONS IN *NAMPT*

Mutations in drug targets represent a well-described mechanism of acquired resistance to anticancer agents, including inhibitors of NAMPT, which multiple groups have described [Table 2]^[23,47-50]^. In one study, HCT-116 (colorectal carcinoma) and NYH (small cell lung carcinoma) cell lines generated to be resistant to FK866 and/or the CHS-828 analog TP201565 were found to harbor several novel heterozygous mutations in *NAMPT*, including H191R, D93del, and Q388R^[47]^. Each of these mutations affected the binding site of FK866 (H191R) or occurred at the dimer interface of NAMPT (D93del and Q388R). To assess whether these mutations were responsible for the observed NAMPT inhibitor resistance, wild-type and *NAMPT* mutants were overexpressed and sensitivity to NAMPT inhibition was assessed. While overexpression of wild-type *NAMPT* resulted in a 20-fold difference in the required dose of FK866 compared to the parental cell line, each mutant required a higher concentration of the drug to be effective. The effect of the H191R mutation was the most potent; the IC_50_ value for the wild-type NAMPT was 110 nM compared to 8,585 nM in the H191R mutant cell line, a nearly 80-fold difference^[47]^. These data suggest that mutations in *NAMPT*, particularly those affecting the binding site, represent a key mechanism of resistance, as changing the shape of the binding pocket may limit relatively large NAMPT inhibitors from gaining access, rendering the drugs inactive. Once mutations like these occur, they make it extremely challenging to increase the effectiveness and potency of the drug.

**Table 2:** Previously reported *NAMPT* mutations associated with NAMPT inhibitor resistance in preclinical models

**Protein change**	**Location of structural change**	**Cancer model**	**Ref.**
H191R	Binding pocket	HCT-116 (colorectal carcinoma)	[47,48]
G217A	Binding pocket	Mia-Paca2 (pancreatic carcinoma)	[49]
G217R	Binding pocket	HCT-116 (colorectal carcinoma); NCI-H520 (squamous cell lung carcinoma)	[49,50]
G217V	Binding pocket	RD (rhabdomyosarcoma)	[49]
Y18C	Binding pocket	HT1080 (fibrosarcoma)	[23]
D93del	Dimer interface	NYH (small cell lung carcinoma); RD (rhabdomyosarcoma)	[47,49]
Q388R	Dimer interface	HCT-116 (colorectal carcinoma)	[47]
S165F	PRPP binding site	RD (rhabdomyosarcoma)	[49]
S165Y	PRPP binding site	NCI-H460 (non-small cell lung carcinoma)	[49]

NAMPT: Nicotinamide phosphoribosyltransferase; PRPP: phosphoribosyl pyrophosphate.

A second study examining a lab-generated FK866-resistant colorectal carcinoma cell line (HCT116R^FK866^) with the H191R mutation demonstrated that this mutation rendered the protein unable to completely form dimers or interact with binding partners such as tPOTEE and beta-actin. This resulted in cross-resistance across the class of NAMPT inhibitors, although the extent of resistance was found to be more related to the head structure of each inhibitor than the linker or tail groups^[48]^. A potential explanation for this is that the head group is the first point of contact with the binding pocket of NAMPT, which is structurally changed in the H191R mutated cells.

An additional study on the colorectal carcinoma cell line HCT-116 with induced resistance to GMX1778 revealed a single point mutation of amino acid 217 from glycine to arginine (G217R)^[50]^. Studies of the crystal structure of the enzyme found that glycine 217 is located near the active site of NAMPT^[51]^. As with the other reported mutations, the amino acid substitution resulted in a change in the structure of the binding pocket, with an arginine at residue 217 creating a steric clash of the arginine side chains with GMX1778 and contributing to the drug resistance. While the G217R mutation had no effect on the catalytic activity of NAMPT, it significantly diminished the sensitivity of NAMPT to GMX1778 inhibition^[50]^.

In addition to the G217R, H191R, and D93del lesions previously described^[47,48,50]^, a study of RD, MiaPaCa-2, NCI-H460, and *NAPRT1* proficient NCI-H520 cell lines identified four novel mutations, G217A, G217V, S165F, and S165Y. Unlike the mutations that changed the structure of the drug-binding pocket, mutations S165F and S165Y resulted in a conformational change in a small helical structural motif spanning the sequence G383GGLLQ388. The expression of S165F/Y mutant NAMPT conferred resistance to GNE-618, demonstrating that mutant NAMPT retained a level of enzymatic activity to support sufficient NAD^+^ production for cell survival^[49]^.

While H191R and G217 mutant cell lines exhibited at least 100-fold increases in GNE-618 IC_50_ compared to wild-type, the effects of the mutations on GMX1778 and FK866 IC_50_ were more diverse, with G217R and H191R inducing the largest changes and G217V and G217A inducing smaller IC_50_ changes^[49]^. The structure of NAMPT may provide an explanation as H191 and G217, together with D219 and Y188, form one side of the tunnel wall surrounding the cavity commonly occupied by NAMPT inhibitors^[21]^. This proximity suggests that these mutations may directly interfere with NAMPT inhibitor binding. An in-silico model predicts that the side chain of H191R would invade the active site tunnel and sterically block inhibitors like FK866 from binding^[47]^. In both the G217A and G217V mutants, the H191 side chain rotates away from the optimal herringbone conformation. This change results in a lesser impact on NAMPT inhibitors with narrow and flexible linkers, such as FK866. In contrast, the G217R mutation resulted in a greater degree of NAMPT inhibitor resistance across the class, likely due to the presence of NAMPT inhibitor binding site changes in addition to the H191 side chain^[49]^. These additional changes suggest that the added structural changes rendered the G217R mutation more deleterious for NAMPT inhibitor binding across structural classes in comparison to its G217A/V counterparts.

Finally, an additional novel mutation of amino acid residue 18 from tyrosine to cysteine (Y18C) has been reported. Overexpression of this mutant in HT1080 fibrosarcoma cells resulted in a substantial decrease in the sensitivity of cells to GMX1778, whereas overexpression of a *NAMPT* Y18F mutant resulted in a smaller change in sensitivity^[23]^. The Y18C mutant likely results in a thiol group on the Y18 gamma-carbon, which is predicted to affect substrate and inhibitor affinity, influencing the modification of later-generation NAMPT inhibitors^[23]^. Collectively, these data indicate that mutations in *NAMPT*, specifically those that affect the structure of the binding site and/or binding pocket or the ability of the protein to interact with binding partners, represent a major potential mechanism of drug resistance to NAMPT inhibitors.

In addition to the *NAMPT* mutations that have been identified preclinically as mediators of acquired resistance to NAMPT inhibitors, recurrent *NAMPT* mutations have been rarely (< 1%) described in patient tumor samples across multiple types of malignancies [Table 3]^[38,52-57]^. These include missense, nonsense, and frameshift deletion mutations. In addition, many non-recurrent mutations in *NAMPT* have also been reported in a variety of cancers [Supplementary Table 1]. It is currently unknown whether these mutations play a role in NAMPT inhibitor drug resistance, as no correlative biology studies have been reported in any of the early-phase NAMPT inhibitor studies^[26-32]^. Future research to determine whether pre-existing or acquired mutations in *NAMPT* may be predictive biomarkers for NAMPT inhibitor activity will be crucial to understanding the functional implications of these genetic changes in patient tumors.

**Table 3:** *NAMPT* mutations in patient tumor specimens that are recurrent across various malignancies

**Protein change**	**Disease type**	**Mutation type**	**Ref.**
K229T	Rectal adenocarcinoma	Missense	[52-55]
	Colorectal adenocarcinoma	Missense	[52-54,56]
A286V	Colon adenocarcinoma	Missense	[52-55]
	Hepatocellular adenoma	Missense	[52-54]
P317H	Astrocytoma	Missense	[52-55]
	Cutaneous melanoma	Missense	[52-55]
G381V	Renal clear cell carcinoma	Missense	[52-54,56]
	Hepatocellular carcinoma	Missense	[52-54,57]
R429*	Renal non-clear cell carcinoma	Nonsense	[52-54]
	Uterine endometrial carcinoma	Nonsense	[52-55]
	Breast invasive ductal carcinoma	Nonsense	[52-55]
K229Nfs*22	Uterine endometrioid carcinoma	Frameshift deletion	[52-55]
	Intestinal-type stomach adenocarcinoma	Frameshift deletion	[52-55]

NAMPT: Nicotinamide phosphoribosyltransferase.

## METABOLIC ADAPTATION

It is well established that cancer cells can undergo metabolic reprogramming to adapt and survive in the presence of cellular stressors, including cancer agents, and multiple such metabolic adaptations have been described in the context of drug resistance^[58]^. One study examining mechanisms of resistance to FK866 in the T-cell leukemia cell line CCRF-CEM and the triple-negative breast cancer cell line MDA MB231 demonstrated that drug resistance was mediated by activation of alternative biochemical pathways that compensated for low NAD^+^ and NADH levels. Specifically, cytosolic ATP production was increased despite a reduction in energy production efficiency of both pathways of the mitochondrial respiratory chain (complex II, III, IV and I, III, IV). This decrease in OXPHOS activity was identified alongside an increase in glucose consumption and lactate production. Furthermore, increased activity of the glycolytic enzymes hexokinase (HK), phosphofructokinase (PFK), pyruvate kinase (PK), and LDH, was observed in resistant cells compared to parental, additionally suggesting an increased reliance on glycolysis. To assess the functional consequences of these changes, parental and resistant cell lines were treated with the LDH inhibitor GSK2837808A (GSK), which slightly re-sensitized the resistant cells to FK866. Silencing *LDHA* with siRNA phenocopied the effect of GSK treatment. Taken together, these data suggest that activation of glycolysis mediates resistance to NAMPT inhibitors^[45]^. Indeed, other studies have shown that combining glycolytic inhibitors, such as FX11, with FK866 results in a synergistic anticancer effect^[59]^, suggesting that combination therapy with inhibitors of glycolysis may represent a rational strategy for preventing or delaying the onset of NAMPT inhibitor resistance.

A second study reported on the role of mitochondrial function as a mechanism of acquired resistance to FK866 in the triple-negative breast cancer cell line MDA-MB-231. Metabolic flux analysis revealed that resistant and parental cells did not exhibit differences in basal glycolysis, glycolytic capacity, or basal OXPHOS, but that resistant cells exhibited a higher mitochondrial spare respiratory capacity^[60]^. This finding suggested a potential role of altered mitochondrial function in the resistant cells. Mitochondrial metabolic plasticity is a known mechanism of resistance to other anticancer therapies, as mitochondrial biogenesis and turnover, fusion and fission are universal mitochondrial stress-adaptive processes^[58,61]^. Indeed, the NAMPT inhibitor-resistant cells exhibited increased expression of *TOMM20*, an indirect measure of mitochondrial mass, *PGC-1α*, a modulator of mitochondrial biogenesis, and *TFAM*, a mitochondrial packaging protein. Confirmatory protein studies showed a 2-fold increase in mitochondrial mass in resistant cells. In addition, resistant cells showed an increase in pyruvic acid oxidation compared to the parental cells, indicating a metabolic shift toward the use of pyruvate as a carbon source for the mitochondria. Pharmacological inhibition of the mitochondrial pyruvate carrier (MPC) with UK5099 resulted in a greater loss of maximal respiration as measured with an extracellular flux assay in the resistant cells than the parental cells, further suggesting that NAMPT inhibitor-resistant cells exhibit a greater dependency on pyruvate for the TCA. Taken together, these data suggest that mitochondrial plasticity in the form of increased mitochondrial mass and dependency on pyruvate for TCA are features of FK866-resistant triple-negative breast cancer cells^[60]^.

## ALTERED DRUG TRANSPORT VIA ATP-BINDING CASSETTE TRANSPORTERS

ATP-binding cassette (ABC) transporters regulate cellular levels of small molecules across cell membranes and have been implicated in the development of drug resistance to anticancer agents, as transporters with broad substrate specificity have the potential to transport multiple chemotherapeutic agents and confer multidrug resistance^[62-64]^. P-glycoprotein (P-GP/ABCB1) is one such transporter that is commonly associated with drug-dependent resistance via increased drug efflux as well as other cellular mechanisms^[65]^.

Only two studies have examined the potential role of P-GP/ABCB1 as a mechanism of resistance to NAMPT inhibitors, yielding contradictory results. In one study, a drug screen testing for active combinations with FK866 in primary leukemia cells identified cyclosporin A, a calcineurin inhibitor and a P-GP/ABCB1 inhibitor, as an enhancer of NAMPT inhibitor activity. Follow-up studies demonstrated that while a second calcineurin inhibitor did not have the same potentiating effect on FK866 in the cells, multiple other P-GP/ABCB1 inhibitors including verapamil and PGP-4008 recapitulated the effect of cyclosporin A on the potency of FK866 in both leukemia cells lines and multiple primary leukemia cells. In addition, the use of cyclosporin A and PGP-4008 increased the concentration of intracellular FK866, lending additional support to the hypothesis that FK866 is a PGP/ABCB1 substrate. Genetic silencing of *P-GP/ABCB1* phenocopied the pharmacologic result with significantly enhanced cell death observed upon FK866 treatment in the *P-GP/ABCB1* knockdown condition^[66]^. Although other interactions between FK866 and the P-GP/ABCB1 inhibitors tested in this study may have contributed to their enhanced combined effects, these results preliminarily suggest that FK866 and possibly other NAMPT inhibitors are substrates of P-GP/ABCB1, that drug transport may be a mechanism of resistance, and that combinations using NAMPT inhibitors plus P-GP/ABCB1 inhibitors may mitigate such resistance.

In the other study, the colorectal carcinoma cell line HCT116 and an FK866-resistant cell line (HCT116R^FK866^) were examined for differences in their transcriptomes. Resistant cells were found to have lower expression of *P-GP*/*ABCB1* transcripts, which was confirmed at the protein level^[67]^. This finding is inconsistent with multiple previous reports demonstrating that overexpression of P-GP/ABCB1 is typically responsible for resistance against anticancer drugs^[68]^. In this study, co-treatment of parental and resistant cells with the P-GP/ABCB1 inhibitor verapamil resulted in increased sensitivity of HCT116R^FK866^ cells, whereas the sensitivity of parental cells was unaffected. The authors concluded that P-GP/ABCB1 may be involved in the efflux of NAMPT inhibitor FK866 in HCT116R^FK866^ cells, although no further mechanistic studies were conducted, limiting the conclusiveness of these findings^[67]^. No other studies have examined the role of drug transporters in NAMPT inhibitor resistance, underscoring the need for additional research in this area.

## CONCLUSION

In summary, several mechanisms of NAMPT inhibitor resistance have been identified, including changes in the expression of the NAD^+^ production enzymes, mutations in the target protein, metabolic reprogramming, and altered expression of ABC efflux transporters (ABCB1). Most of these mechanisms have been identified using a small number of cell lines representing an even smaller number of cancer types, and therefore other unidentified mechanisms of resistance are likely. Additional work is necessary to better understand how the biology of a given cancer cell may predispose that cell to resistance. In addition, the mechanisms underlying these changes remain unclear - for example, whether they arise through a process of selection or adaptation. A deeper understanding of these mechanisms is critical so that strategies to bypass them can be identified and will contribute to the further development of this class of agents.

## DECLARATIONS

### Copyright

© The Author(s) 2025.
